# *Cacna1c* deficiency in forebrain glutamatergic neurons alters behavior and hippocampal plasticity in female mice

**DOI:** 10.1038/s41398-024-03140-2

**Published:** 2024-10-06

**Authors:** Srivaishnavi Loganathan, Danusa Menegaz, Jan Philipp Delling, Matthias Eder, Jan M. Deussing

**Affiliations:** 1 Research Group Molecular Neurogenetics, Max Planck Institute of Psychiatry, Munich, Germany; 2 International Max Planck Research School for Translational Psychiatry (IMPRS-TP), Munich, Germany; 3 Scientific Core Unit Electrophysiology, Max Planck Institute of Psychiatry, Munich, Germany; 4 Research Group Neural Dynamics and Behavior, Max Planck Institute of Psychiatry, Munich, Germany

**Keywords:** Hippocampus, Psychiatric disorders

## Abstract

*CACNA1C*, coding for the α1 subunit of L-type voltage-gated calcium channel (LTCC) Ca_v_1.2, has been associated with multiple psychiatric disorders. Clinical studies have revealed alterations in behavior as well as in brain structure and function in *CACNA1C* risk allele carriers. These findings are supported by rodent models of Ca_v_1.2 deficiency, which showed increased anxiety, cognitive and social impairments as well as a shift towards active stress-coping strategies. These behavioral alterations were accompanied by functional deficits, such as reduced long-term potentiation (LTP) and an excitation/inhibition (E/I) imbalance. However, these preclinical studies are largely limited to male rodents, with few studies exploring sex-specific effects. Here, we investigated the effects of Ca_v_1.2 deficiency in forebrain glutamatergic neurons in female conditional knockout (CKO) mice. CKO mice exhibited hyperlocomotion in a novel environment, increased anxiety-related behavior, cognitive deficits, and increased active stress-coping behavior. These behavioral alterations were neither influenced by the stage of the estrous cycle nor by the *Nex/Neurod6* haploinsufficiency or Cre expression, which are intrinsically tied to the utilization of the *Nex-Cre* driver line for conditional inactivation of *Cacna1c*. In the hippocampus, Ca_v_1.2 inactivation enhanced presynaptic paired-pulse facilitation without altering postsynaptic LTP at CA3-CA1 synapses. In addition, CA1 pyramidal neurons of female CKO mice displayed a reduction in dendritic complexity and spine density. Taken together, our findings extend the existing knowledge suggesting Ca_v_1.2-dependent structural and functional alterations as possible mechanisms for the behavioral alterations observed in female *Ca*_*v*_*1.2-Nex* mice.

## Introduction

*CACNA1C*, encoding the α1 subunit of the L-type voltage-gated calcium channel (LTCC) Ca_v_1.2, is one of the most robust and consistently replicated genetic risk factors for multiple psychiatric disorders including schizophrenia (SCZ), bipolar disorder (BPD), major depressive disorder (MDD), autism spectrum disorder (ASD) and attention-deficit hyperactivity disorder (ADHD) [1, 2]. Disease-associated *CACNA1C* single nucleotide polymorphisms (SNPs), like the well-studied rs1006737, have been associated with behavioral as well as structural and functional alterations of the central nervous system [3, 4]. Risk allele carriers have been shown to exhibit, among other anomalies, deficits in cognitive performance, age-related cortical thinning, and reduced activation of different brain regions [5–8]. Gene expression analysis in post-mortem brain tissue or fibroblast-derived neurons of risk allele carriers revealed altered *CACNA1C* mRNA expression levels, further supporting its implication in the pathology of psychiatric disorders [9–12].

The LTCC family consists of four distinct members: Ca_v_1.1 - Ca_v_1.4, among which Ca_v_1.2 and Ca_v_1.3 are predominant in the brain, with Ca_v_1.2 accounting for 89% of LTCCs [4]. In neurons, Ca_v_1.2 is localized postsynaptically at dendrites and at the soma, where it critically regulates protein synthesis-dependent late-phase LTP [13, 14]. Calcium influx through Ca_v_1.2 leads to post-depolarization events including activation of Ca^2+^/calmodulin-dependent protein kinase II (CaMKII), mitogen-activated protein kinase (MAPK)/extracellular signal-regulated kinase (ERK), and calcineurin/nuclear factor of activated T-cells (NFAT) pathways and excitation-transcription coupling [4]. These pathways are important for driving late-phase LTP and are crucially involved in dendritic development, neuronal survival, synaptic plasticity, memory formation, and behavior [4, 13]. In addition, presynaptic localization has also been reported, with Ca_v_1.2 being involved in the maintenance of presynaptic LTP [15–17].

For many psychiatric disorders, including MDD, post-traumatic stress, and anxiety disorders, it is well-documented that females show a higher disease prevalence than males. Along these lines, sex-specific effects have also been reported for disease-associated *CACNA1C* SNPs, with female risk allele carriers showing, e.g., impaired recovery from schizophrenia-spectrum episodes, greater hostility, and harm avoidance as well as increased frequency of paranoia [18, 19]. However, past preclinical research has largely focused on male rodents, which is, to some extent, owing to the efforts to minimize variability potentially arising from hormonal fluctuations connected to the estrous cycle. Accordingly, preclinical studies using male Ca_v_1.2 rodent models have consistently reported increased anxiety-related behavior, impaired social behavior, and cognitive deficits [2, 20]. Previous studies from our lab also revealed alterations of psychiatric disorder-related endophenotypes which differentially manifest depending on the timepoint (embryonic versus adulthood) of Ca_v_1.2 deletion in forebrain glutamatergic neurons [21]. In addition to behavioral alterations, Ca_v_1.2 deficiency in different neuronal populations has been shown to alter protein synthesis, excitation/inhibition (E/I) balance, LTP, and neurogenesis [21–24]. Moreover, Ca_v_1.2 has been demonstrated to directly affect the morphology of neurons. For example, gain-of-function mutations promote dendritic retractions in vitro and in vivo [25], while calcium influx through Ca_v_1.2 is involved in synaptic pruning in medium spiny neurons [25, 26]. Taken together, previous studies revealed a vital role for Ca_v_1.2 in modulating different behaviors in male rodents and were able to shed light on potential underlying mechanisms.

Nevertheless, extending these findings beyond the current gender bias by incorporating data from female rodents is essential to advance our comprehension of Ca_v_1.2 function in health and disease. In this study, we thus focused on female mice lacking *Cacna1c* in forebrain glutamatergic neurons to identify potential sex-specific effects of Ca_v_1.2 on behavior as well as functional and structural alterations in the hippocampus.

## Materials and methods

### Animals

The *Ca*_*v*_*1.2-Nex* mouse line lacking *Cacna1c* in forebrain glutamatergic neurons was generated by breeding *Cacna1c*^*lox/lox*^ mice (*Cacna1c*^*tm3Hfm*^) to *Nex-Cre* (*Neurod6*^*tm1(cre)Kan*^) mice resulting in control (Ctrl; *Cacna1c*^*lox/lox*^*)* and conditional knockout (CKO; *Cacna1c*^*lox/lox*^*::Nex-Cre*) mice as previously described [21]. To evaluate the recombination pattern of the utilized Cre driver line, we bred *Nex-Cre* mice to *RiboTag mice* (*Rpl22*^*tm1.1Psam*^) generating *RiboTag-Nex* mice. In *RiboTag* mice the ubiquitously expressed ribosomal protein L22 (RPL22) is equipped with a Cre recombinase-dependent hemagglutinin epitope (HA) tag. Cre mediates a substitution of wild-type RPL22 by the HA-tagged variant which can readily be detected using an HA-specific antibody [27]. Hence, we used *RiboTag-Nex* mice as a reporter to visualize the pattern and penetrance of Cre recombination. Female mice at 4–6 months of age were used for all behavioral experiments. Separate cohorts of female mice were used for the Morris water maze test (6 months of age) and for the Golgi-Cox staining (10 months of age). To control for potential unspecific Cre effects, 4–6 months heterozygous and wild-type *Nex-Cre* mice were subjected to behavioral testing [28]. Mice were group-housed (single-housed only for home cage activity recordings) under standard laboratory conditions (22 ± 1 °C, 55 ± 5% humidity) and maintained on a 12-h light-dark cycle with *ad libitum* food and water. All animal experiments were conducted in accordance with and approved by the Guide for the Care and Use of Laboratory Animals of the Government of Upper Bavaria, Germany.

### Estrous cycle determination

The estrous cycle was monitored for two weeks prior to behavioral assessment and after every behavioral test. Vaginal lavages were collected in the afternoon between 3:00 pm and 5:00 pm according to previously described protocols [29, 30]. Briefly, 1× phosphate-buffered saline (PBS) was aspirated using a pipette on the mouse vaginal canal until the solution turned cloudy, smeared on a glass slide, and allowed to dry at 37 °C for 30 min. The smears were stained with Wright–Giemsa stain (Sigma, #WG16-500ML) and the four stages of the estrous cycle stages were determined according to previously described criteria: proestrous, round and clustered small and large nucleated epithelial cells with cells appearing violet in color; estrous, densely packed clusters of anucleated cornified squamous epithelial cells in blue/light blue color; metestrous, small leukocytes and cornified squamous epithelial cells appearing dark purple; diestrous, leukocytes along with nucleated epithelial cells and very few cornified squamous epithelial cells appearing dark purple in color [30].

### Behavioral analysis

Behavioral characterization of *Ca*_*v*_*1.2-Nex* and *Nex-Cre* mice was performed between 9:00 am and 3:00 pm. All tests were performed by an experienced, blinded researcher and according to established protocols. Animals were allocated to the experimental groups according to their genotype in a semi-randomized manner and data analysis was performed blinded to the group allocation. Vaginal lavages from *Ca*_*v*_*1.2-Nex* mice for estrous cycle determination were obtained at the end of each behavioral test except for home cage activity and Morris water maze. Behavioral experiments were recorded and analyzed using ANY-Maze software (Stoelting Co., Wood Dale, Illinois, USA) unless otherwise mentioned.

#### Home cage activity

Animals were single-housed for the home cage activity measurements. Home cage activity was monitored using an automated infrared tracking system for 7 days (Mouse-E-Motion 2.3.6, Infrared-E-Motion, Hagendeel, Germany). The activity data were analyzed from day 3 (after a 2-day habituation period) for 96 h to obtain accurate measures of activity during both light and dark cycles.

#### Open field test (OFT)

Locomotor activity in a novel environment was investigated in the OFT. Testing was performed in a dimly lit (<30 lux) open field arena where mice were allowed to explore the apparatus freely for 30 min. Total distance traveled, time spent in the inner zone, and number of inner zone entries were assessed with the ANY-maze software.

#### Elevated plus maze (EPM)

Anxiety-related behavior was investigated in the EPM. Testing was conducted in a plus-shaped elevated maze where mice were allowed to explore freely for 10 min and total distance traveled, time spent in the open arms, and a number of open arm entries were assessed using the ANY-maze software.

#### Y-maze spontaneous alternations test

To test working memory, mice were allowed to freely explore the apparatus for 10 min. The number of triads (for example: ABC, BCA, CAB…) and the total number of arm entries were scored manually, and the percentage of spontaneous alternations was calculated accordingly.

#### Morris water maze (MWM)

Spatial memory was assessed using the classical MWM. Mice were trained to find a submerged platform on 5 consecutive days (4 trials per day with each trial lasting 90 s and an intertrial interval of 20 min). Average latencies to reach the platform were calculated. A probe trial was conducted 24 h and 7 days after the last trial of the last training day to determine short-term and long-term memory. Time spent in each quadrant was calculated.

#### Forced swim test (FST)

The FST was performed to assess active versus passive stress-coping behavior. Each mouse performed the test for 6 min and times spent struggling, swimming, and floating were scored manually.

### Electrophysiological recordings

8–12 week-old mice were anesthetized with isoflurane and decapitated. The brain was rapidly removed from the cranial cavity and, using a vibratome (HM650V, Thermo Scientific), 350 µm-thick coronal slices containing the dorsal hippocampus were cut in an ice-cold carbogen gas (95% O_2_/5% CO_2_)-saturated solution consisting of (in mM): 87 NaCl, 2.5 KCl, 25 NaHCO_3_, 1.25 NaH_2_PO_4_, 0.5 CaCl_2_, 7 MgCl_2_, 10 glucose, and 75 sucrose. Slices were incubated in carbogenated physiological saline for 30 min at 34°C and, afterward, for at least 30 min at room temperature (23–25 °C). The physiological saline contained (in mM): 125 NaCl, 2.5 KCl, 25 NaHCO_3_, 1.25 NaH_2_PO_4_, 2 CaCl_2_, 1 MgCl_2_, and 10 glucose. All measurements were conducted at room temperature. During field potential recordings, slices were superfused with carbogenated physiological saline (4–5 ml/min flow rate).

Whole-cell patch-clamp recordings from CA1 pyramidal neurons were performed as previously described [13]. The carbogenated extracellular solution (2–3 ml/min flow rate) contained (in mM): glucose 10, CaCl_2_ 2, MgCl_2_ 1, TEA-Cl, KCl 3, NaH_2_PO_4_ 1.25, NaHCO_3_ 26 and TTX 0.001. Patch pipettes (3–5 MΩ open-tip resistance) were filled with a solution consisting of (in mM): Cs gluconate 120, TEA-Cl 20, EGTA 1, ATP magnesium salt 4, GTP sodium salt 0.4 and HEPES 10 (pH 7.4). Inward currents were evoked by voltage-clamp ramps from –80 mV to +80 mV with 0.5 mV/ms, while the holding potential was –70 mV. The selective loss of L-type Ca^2+^ currents in CKO mice was determined by the application of the L-type channel blocker isradipine (10 µM).

Field excitatory postsynaptic potentials (fEPSPs) at CA3-CA1 synapses were evoked by square-pulse electrical stimuli (50 µs pulse width) delivered via a bipolar tungsten electrode (50 μm pole diameter, ∼0.5 MΩ nominal impedance) to the Schaffer collateral-commissural pathway. fEPSPs were recorded using glass microelectrodes (filled with physiological saline, ~1 MΩ open-tip resistance) that were placed into the CA1 stratum radiatum. The intensity of voltage stimulation was adjusted in a manner to produce a fEPSP of ∼50% of the amplitude at which a population spike appeared. Recording data were low-pass filtered at 1 kHz and digitized at 5 kHz. Before and after LTP induction, a single stimulation pulse was delivered every 15 s to the neural tissue. LTP was induced by high-frequency stimulation (HFS, 100 Hz for 1 s). Paired-pulse ratio was calculated by dividing the slope of fEPSP2 by the slope of fEPSP1.

### Immunohistochemistry

For immunohistochemistry on frozen tissue sections, animals were sacrificed using isoflurane and subsequently perfused with ice-cold 1× PBS and 4% PFA. Dissected brains were post-fixed in 4% PFA overnight at 4 °C, transferred to 30% sucrose in 1× PBS, and incubated at 4 °C for 48 h. Brains were frozen on dry ice and cut coronally in 40 µm sections using a cryostat (Leica). Sections were collected in cryoprotection solution (25% Ethylene glycol, 25% glycerol, 50% ddH_2_O in 1× PBS) and stored at –20 °C until further use. For immunostaining, slices were washed 3× in 1× PBS, followed by blocking in 2% normal goat serum in 0.05% Triton-X100 and 1× PBS. Sections were incubated with primary antibody against HA-tag (Cell Signaling #C29F4, 1:1000) at 4 °C overnight. After washing, sections were incubated with a secondary antibody (goat anti-rabbit Alexa Fluor™ Plus 488, ThermoFisher Scientific #A32731, 1:1000) for 2 h at room temperature. Finally, sections were washed and mounted using Fluoromount-G mounting medium with DAPI (Southern Biotech, #0100-20). Images were acquired using Olympus SlideScanner VS120S6.

### Golgi-Cox staining

Fresh brain tissue was harvested from mice and the Golgi-Cox procedure was performed using the Bioenno superGolgi kit according to the manufacturer´s instructions (Catalog #003010, Bioenno Tech, CA, USA). Briefly, freshly harvested brain tissues were incubated in solution A (impregnation solution) for 14 days followed by incubation in solution B for 3 days. Brain tissues were cut at 150 µm thickness, mounted on gelatin-coated slides, and air-dried overnight. Sections were stained, dehydrated, cover-slipped, and stored in the dark at room temperature until imaging. Dendritic arbors and spines were visualized and traced using a bright field microscope (Zeiss Axio Imager M2) and Neurolucida software (v.2017, MicrobrightField, USA). Neurons from the CA1 hippocampal region were selected if at least 3 completely stained dendritic trees (including the apical tree) were visible and the apical dendritic tree was not broken or incomplete. Dendritic arbors were traced with 40× objective with the Neurolucida software. Spines on secondary and tertiary branches were traced with 100× oil objective for spine density analysis. Data from Sholl, branched structure, and dendritic segment analyses were obtained from Neurolucida Explorer 2017. Spine density was calculated accordingly.

### Statistical analysis

Statistical analyses were performed using the commercially available GraphPad Prism v7.0 (GraphPad Software, La Jolla, CA, USA). The sample size was chosen such that with a type 1 error of 0.05 and a type 2 error of 0.2, the effect size should be at least 1.2-fold of the pooled standard deviation. All results are presented as mean ± s.e.m. Estimation of variation within each group of data revealed a similar degree of variation in the statistically compared groups. Student’s t-test (two-tailed) was used to evaluate behavioral and morphological phenotypic differences between the two genotypes. For time-dependent measures and Sholl analysis, a two-way analysis of variance (ANOVA) with repeated measures was used. For paired-pulse facilitation analysis, two-factorial ANOVA was used. Whenever significant main or interaction effects were found, Bonferroni *post hoc* tests were carried out to identify simple effects. Statistical significance was defined as p < 0.05. All data were tested for outliers using Grubbs’ test.

## Results

### Ca_v_1.2 deficiency promotes hyperactivity and increases anxiety-related behavior

**Fig. 1:**
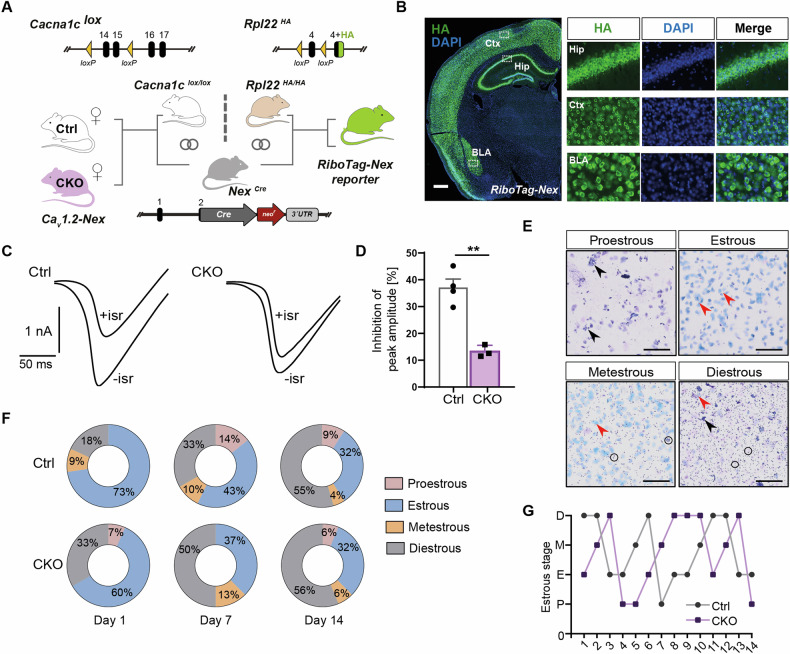
Ca_v_1.2 deficiency in forebrain glutamatergic neurons does not alter the estrous cycle. **A** Scheme illustrating the generation of the *Ca*_*v*_*1.2-Nex* mouse line (left) and *RiboTag-Nex* reporter mice (right) by breeding *Nex-Cre* mice to *Cacna1c*^*lox*^ or *Rpl22*^*HA*^ (RiboTag) mice, respectively. **B** Coronal brain section and higher magnifications of *RiboTag-Nex* reporter mouse illustrating the Cre-dependent recombination in glutamatergic neurons of hippocampus (Hip), cortex (Ctx), and basolateral amygdala (BLA). **C** Representative examples of whole-cell Ca^2+^ currents evoked by voltage-clamp ramps from –80 mV to +80 mV (0.5 mV/ms) in hippocampal CA1 pyramidal neurons from Ctrl and CKO mice before (–isr) and after (+isr) application of the L-type channel blocker isradipine (10 µM). **D** The reduction of the peak inward current in CA1 pyramidal neurons from control (n = 4 cells from 2 mice) and CKO (n = 3 cells from 2 mice) mice. Data are means ± S.E.M. (**p = 0.0018). **E** Representative micrographs of different stages of the estrous cycle (Scale bar: 100 µm). Black arrows indicate nucleated epithelial cells, red arrows indicate anucleated cornified squamous epithelial cells and circles indicate leukocytes. **F** Percentage of animals in each stage of the estrous cycle on days 1, 7, and 14**. G** Representative graph showing the lengths of different estrous cycle stages in Ctrl and CKO mice (n = 1 per group, randomly selected).

Female mice lacking Ca_v_1.2 in forebrain glutamatergic neurons were generated by crossing *Cacna1c*^*lox/lox*^ mice to *Nex-Cre* mice (Fig. 1A). The *Nex-Cre* driver line conveys extensive and complete recombination in forebrain glutamatergic neurons in the cortex, hippocampus, and basolateral amygdala (Fig. 1B). To demonstrate the loss of functional Ca_v_1.2 channels, we followed the strategy of Moosmang and colleagues [13] and assessed the dihydropyridine (DHP)-sensitive Ca^2+^ inward currents evoked by voltage-clamp ramps. Addition of the DHP isradipine (10 μM) to the bath solution significantly decreased the Ca^2+^ current amplitude in CA1 pyramidal neurons from Ctrl mice while it had only a minor effect on Ca^2+^ currents in CA1 pyramidal neurons from CKO mice (Fig. 1C). On average, the portion of the peak Ca^2+^ inward current inhibited by isradipine was 37.09 ± 2.85% (Ctrl; n = 4 from 2 mice) and 13.29 ± 1.13% (CKO; n = 3 neurons from 2 mice). This is equivalent to a 64% reduction of the DHP-sensitive current in CA1 pyramidal neurons of the mutant mice demonstrating the efficient deletion of CACNA1C from forebrain glutamatergic neurons (Fig. 1D).

**Fig. 2:**
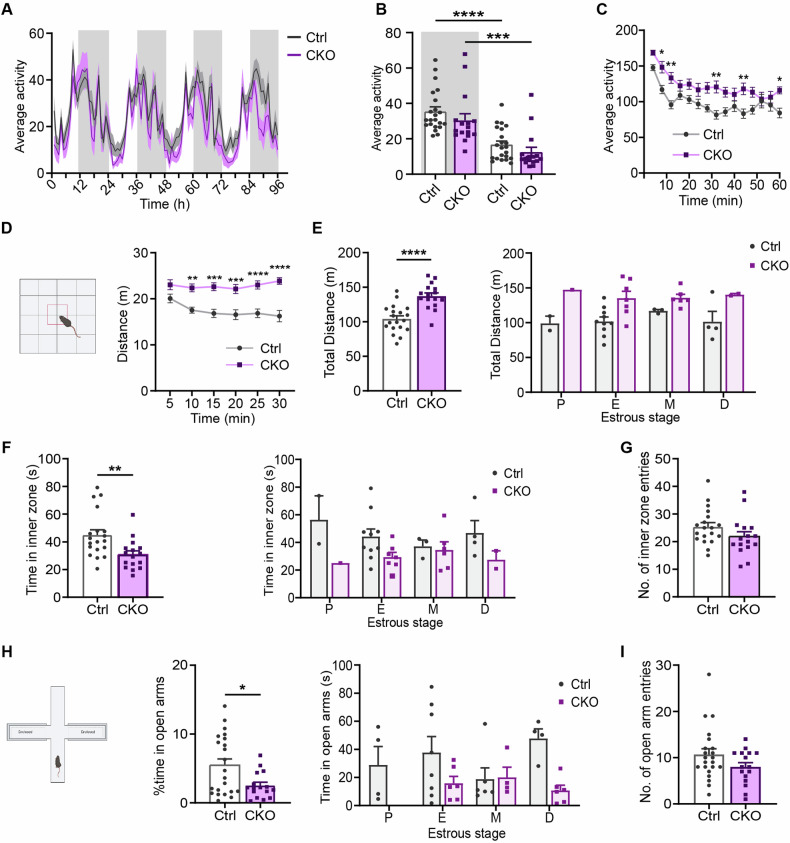
Ca_v_1.2 deficiency promotes hyperlocomotion and increases anxiety-related behavior. **A** Average activity of female *Ca*_*v*_*1.2-Nex* mice in a novel home cage monitored for 96 h. The dark phase is indicated by gray-shaded areas. **B** Average basal home cage activity during night (shaded) and day. **C** Average activity in the first 60 min following introduction into a novel home cage. **D** Distance traveled in the OFT split into 5-min time bins. **E** Collapsed total distance traveled in 30 min and separated according to the different estrous stages. **F** Collapsed time spent in the inner zone of the OFT during the first 5 min of the test and split according to different estrous stages. **G** Number of inner zone entries during the first 5 min of the OFT. **H** Collapsed percentage of time spent in the open arms of the EPM and detailed according to different estrous stages. **I** Number of open arm entries in the EPM (Ctrl: n = 22, CKO: n = 16). Data are represented as mean ± S.E.M., *p < 0.05, **p < 0.01, ***p < 0.001 and ****p < 0.0001. P proestrous. E estrous, M metestrous, D diestrous.

First, we monitored the estrous cycle of female *Ca*_*v*_*1.2-Nex* mice for two weeks. Ctrl and CKO animals showed comparable lengths of different estrous stages (Fig. 1E–G). To assess general activity patterns and circadian rhythmicity, we recorded the home cage activity of *Ca*_*v*_*1.2-Nex* mice following single housing in a novel cage for one week. Over the entire time period, female CKO mice showed activity levels similar to their Ctrl littermates [home cage locomotion: two-way ANOVA; interaction, F_(95, 3420)_ = 1.144, p = 0.1633; genotype, F_(1, 36)_ = 1.697, p = 0.2010; time, F_(95, 3420)_ = 15.28, p < 0.0001] (Fig. 2A). As expected of nocturnal animals, Ctrl and CKO mice showed higher activity during the night than during the day [one-way ANOVA, F_(3, 72)_ = 16.66, p < 0.0001] (Fig. 2B). Interestingly, female CKO mice showed hyperactivity in the first 60 min after being introduced into the novel cage [two-way ANOVA; interaction, F_(14, 504)_ = 1.562, p = 0.0857; genotype, F_(1, 36)_ = 15.75, p = 0.0003; time, F_(14, 504)_ = 15.09, p < 0.0001] (Fig. 2C), suggesting that Ca_v_1.2 deficiency in forebrain glutamatergic neurons induces hyperlocomotion in an unfamiliar environment.

**Fig. 3:**
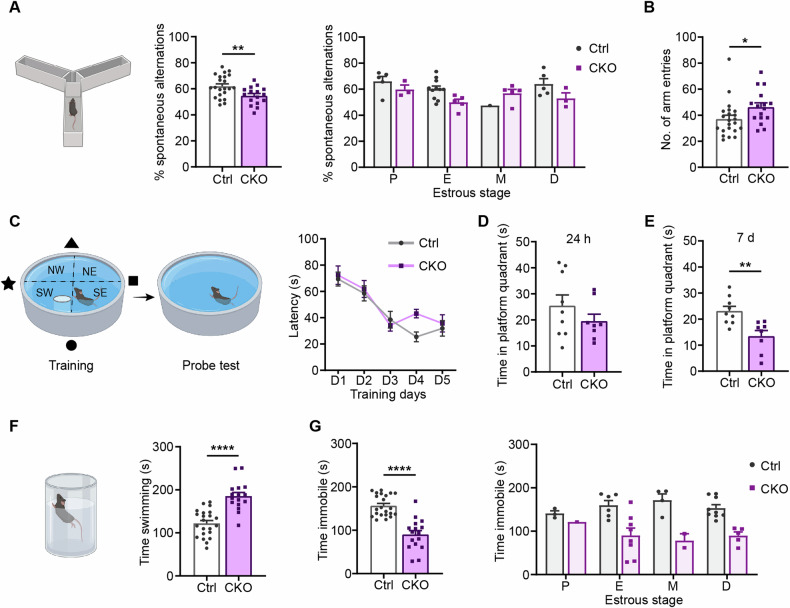
Ca_v_1.2 deficiency is accompanied by cognitive deficits and enhanced active stress-coping. **A** Collapsed percentage of spontaneous alternations of *Ca*_*v*_*1.2-Nex* mice in the Y-maze and split according to different estrous stages. **B** Number of arm entries in the Y-maze. **C** Average latency to finding the submerged platform in MWM. Time spent in target quadrant during the probe test, (**D**) 24 h, and (**E**) 7 days after the last training day. **F** Swimming time of *Ca*_*v*_*1.2-Nex* mice in the FST. **G** Collapsed immobility time in FST and split according to different estrous stages. (For Y-maze and FST - Ctrl: n = 22, CKO: n = 16; for MWM - Ctrl: n = 9, CKO = 8). Data are represented as mean ± S.E.M., *p < 0.05, **p < 0.01, and ****p < 0.0001.

Next, we subjected *Ca*_*v*_*1.2-Nex* mice to a series of behavioral tests to assess locomotion, anxiety-related behavior, cognitive performance, and stress-coping strategies (Figs. 2D–I and 3). In agreement with the first 60 min of the previous long-term home cage activity measurement, CKO mice showed hyperactivity in the 30 min OFT throughout the experiment [two-way ANOVA-repeated measures, interaction, F_(5,165)_ = 3.077, p = 0.0111; genotype, F_(1, 33)_ = 25.99, p < 0.0001; time, F_(5, 165)_ = 3.004, p = 0.0127] (Fig. 2D), resulting in an increased distance traveled compared to their Ctrl littermates [student’s t-test, total distance: t_33_ = 5. 098, p < 0.0001] (Fig. 2E). Furthermore, female CKO mice spent less time in the inner zone of the OFT apparatus during the first 5 min of the task compared to their Ctrl littermates [student’s t-test, inner zone time: t_33_ = 2.931, p = 0.0061] (Fig. 2F), while both groups showed similar numbers of entries to the inner zone [student’s t-test, inner zone entries: t_33_ = 1.546, p = 0.1317] (Fig. 2G). In the EPM, CKO mice spent less time in the open arms [student’s t-test, open arm time: t_36_ = 2.564, p = 0.0147] (Fig. 2H), while the number of entries to the open arms was unaffected [student’s t-test, open arm entries: t_36_ = 1.63, p = 0.1118] (Fig. 2I). These findings suggest that Ca_v_1.2 deficiency in glutamatergic neurons promotes hyperactivity in a novel environment and increases anxiety-related behavior in female mice. Of note, the behavioral phenotypes observed in the OFT and EPM were independent of the different stages of the estrous cycle [two-way ANOVA; OFT distance: interaction, F_(3, 27)_ = 0.4715, p = 0.7046; genotype, F_(1, 27)_ = 15.64, p = 0.0005; estrous stage, F_(3, 27)_ = 0.2733, p = 0.8441, OFT inner zone time: interaction, F_(3, 27)_ = 0.7439, p = 0.5353; genotype, F_(1, 27)_ = 6.929, p = 0.0139; estrous stage, F_(3, 27)_ = 0.07102, p = 0.9750, EPM open arm time: genotype, F_(1, 33)_ = 6.775, p = 0.0137; estrous stage, F_(3, 33)_ = 0.541, p = 0.6572] (Fig. 2E, F, H).

### Ca_v_1.2 deficiency results in hippocampus-related cognitive deficits

Cognitive deficits are among the most robust previously described phenotypes of Ca_v_1.2 mouse models. Therefore, we assessed learning and memory in *Ca*_*v*_*1.2-Nex* female mice. In the Y-maze, female CKO mice showed a significantly reduced number of spontaneous alternations and higher total arm entries compared to their Ctrl littermates [student’s t-test, %alternations: t_36_ = 2.796, p = 0.0083; arm entries: t_36_ = 2.08, p = 0.0447] (Fig. 3A, B). The observed working memory deficit was independent of the different stages of the estrous cycle [two-way ANOVA: interaction, F_(3, 30)_ = 1.715, p = 0.1849; genotype, F_(1, 30)_ = 2.273, p = 0.1421; estrous stage, F_(3, 30)_ = 2.393, p = 0.0881] (Fig. 3A).

In addition, female *Ca*_*v*_*1.2-Nex* mice were subjected to the MWM to assess their spatial learning capabilities. Ctrl and CKO mice learned the task equally well, as reflected by the similar latencies to find the platform during the training days [two-way ANOVA-repeated measures: interaction, F_(4, 60)_ = 0.903, p = 0.4680; genotype, F_(1, 15)_ = 3.428, p = 0.0839; time, F_(4, 60)_ = 16.87, p < 0.0001] (Fig. 3C). We also found that Ctrl and CKO mice spent similar time in the platform quadrant in the probe trial 24 h after the last training day [student’s t-test, t_15_ = 1.177, p = 0.2574] (Fig. 3D). However, when these mice were subjected to a probe trial 7 days after the last training day, CKO mice spent significantly less time in the platform quadrant compared to their Ctrl littermates [student’s t-test, t_15_ = 3.526, p = 0.0031] (Fig. 3E). Taken together, our data suggest that Ca_v_1.2 deficiency in forebrain glutamatergic neurons leads to working memory and hippocampus-related deficits in spatial memory in female mice.

### Ca_v_1.2 deficiency promotes an active stress-coping strategy

Another phenotype previously reported in *Ca*_*v*_*1.2-Nex* male mice was the enhanced active stress-coping behavior in the FST [21]. Thus, we also assessed stress-coping behavior in female *Ca*_*v*_*1.2-Nex* mice. In line with previous findings, female CKO mice showed enhanced active stress-coping behavior in the FST compared to their Ctrl littermates, which was reflected by increased swimming (Fig. 3F) [student’s t-test, time swimming: t_36_ = 5.971, p = <0.0001] and reduced immobility time (Fig. 3G) [student’s t-test, time immobile: t_36_ = 6.744, p < 0.0001]. Also, the alterations in stress-coping behavior were independent of the stages of the estrous cycle [two-way ANOVA: interaction, F_(3, 30)_ = 0.9279, p = 0.4393; genotype, F_(1, 30)_ = 23.32, p < 0.0001; estrous stage, F_(3, 30)_ = 0.08340, p = 0.9686] (Fig. 3G).

### Neither *Nex/Neurod6* haploinsufficiency nor Cre expression affects behavior

**Fig. 4:**
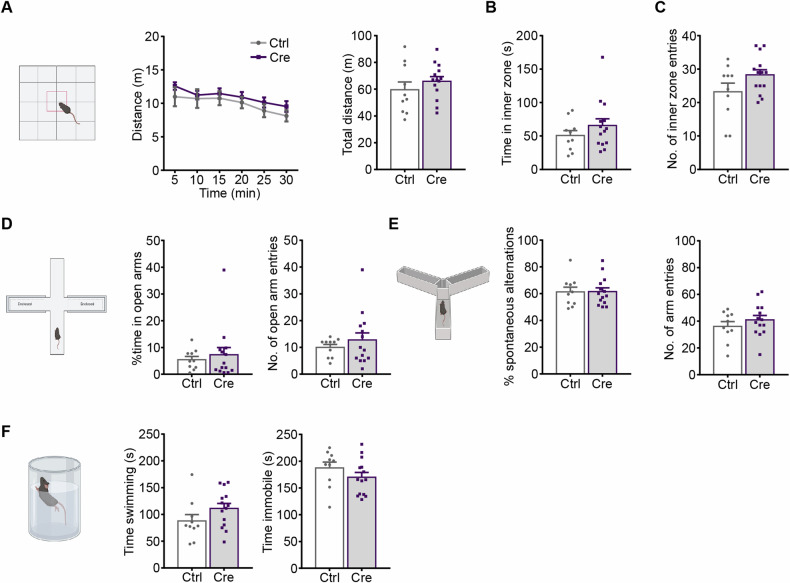
Neither *Neurod6/Nex* heterozygosity nor Cre expression affects behavior in female *Nex-Cre* mice. **A** Distance traveled by *Ca*_*v*_*1.2-Nex* mice in the OFT split in 5-min time bins and total distance traveled throughout 30 min of the test. **B** Time spent in the inner zone and **C** number of entries to the inner zone of the OFT. **D** Percentage of time spent in open arms and number of open arm entries in the EPM. **E** Percentage of spontaneous alternations and number of arm entries in the Y-maze. **F** Time spent swimming and immobile in the FST (Ctrl: n = 10, Cre: n = 14). Data are represented as mean ± S.E.M.

*Nex-Cre* mice were generated by a knock-in strategy, thus heterozygous Cre carriers lack one functional *Nex/Neurod6* allele [28]. To verify that the behavioral phenotypes observed in *Ca*_*v*_*1.2-Nex* female mice were a consequence of the deficiency of Ca_v_1.2 in forebrain glutamatergic neurons and not a result of heterozygous disruption of the *Nex/Neurod6* locus or Cre recombinase expression, we tested the *Nex-Cre* driver mouse line in the same behavioral test battery as *Ca*_*v*_*1.2-Nex* mice. Compared to the Ctrl littermates, heterozygous *Nex-Cre* female mice (*Neurod6*^*+/Cre*^) did not show any significant differences in the OFT [two-way ANOVA-repeated measures, locomotion: interaction, F_(5, 110)_ = 0.3286, p = 0.8947; genotype, F_(1, 22)_ = 0.9508, p = 0.3401; time, F_(5, 110)_ = 8.399, p < 0.0001; student’s t-test, total distance: t_22_ = 0.9751, p = 0.3401; inner zone time: t_22_ = 1.121, p = 0.2743; inner zone entry: t_22_ = 1.804, p = 0.085] (Fig. 4A–C) or EPM [student’s t-test open arm time: t_22_ = 0.5497, p = 0.588; open arm entry: t_22_ = 0.8544, p = 0.4021] (Fig. 4D). In addition, no significant differences were observed in the Y-maze [student’s t-test, spontaneous alternations: t_22_ = 0.05155, p = 0.9594; arm entries: t_22_ = 0.9759, p = 0.3397] (Fig. 4E) or FST [swimming time: student’s t-test, t_22_ = 1.564, p = 0.132; immobility time: student’s t-test, t_22_ = 1.248, p = 0.2253] (Fig. 4F). Of note, no altered behavior was observable in heterozygous *Nex-Cre* male mice compared to the Ctrl littermates in any of the above-mentioned behavioral tests (Fig. S1). In sum, these results indicate that the behavioral phenotypes observed in *Ca*_*v*_*1.2-Nex* female CKO mice are exclusively driven by the loss of Ca_v_1.2 in forebrain glutamatergic neurons and not affected by the *Nex/Neurod6* haploinsufficiency or Cre recombinase expression.

### Ca_v_1.2 inactivation does not alter LTP but reduces neurotransmitter release probability

**Fig. 5:**
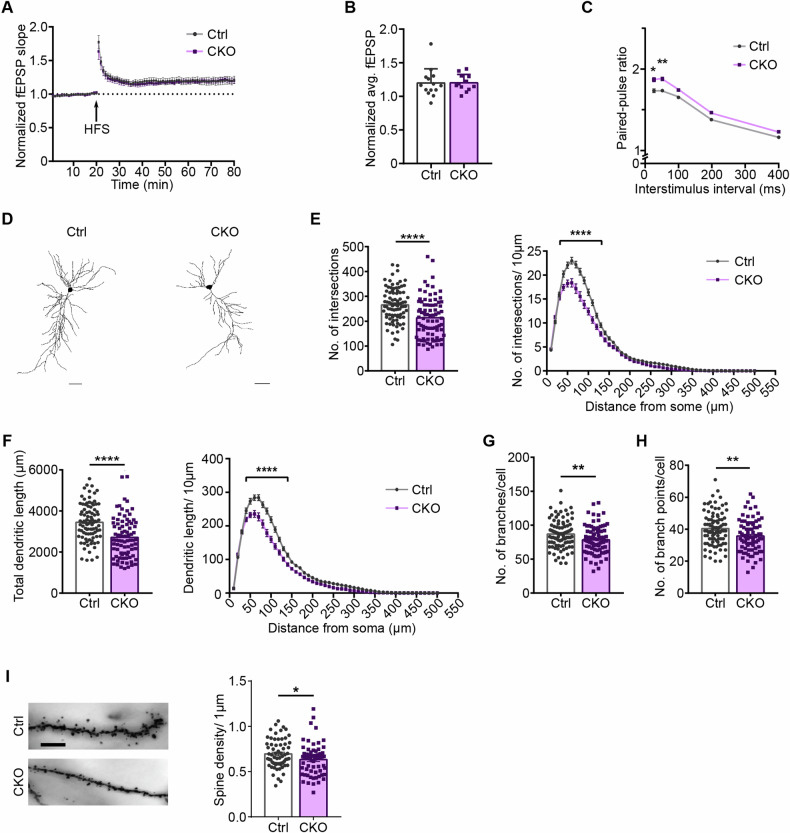
Ca_v_1.2 deficiency alters short-term synaptic plasticity and causes structural alterations in the hippocampus. **A** Normalized field excitatory postsynaptic potential (fEPSP) slope assessed in *Ca*_*v*_*1.2-Nex* mice. **B** Normalized average fEPSP in the last 10 min of LTP recordings in Ctrl and CKO mice. **C** Assessment of paired-pulse ratio reveals enhanced paired-pulse facilitation in *Ca*_*v*_*1.2-Nex* mice (LTP recordings, Ctrl: n = 14 slices from 4 animals, CKO: n = 12 slices from 4 animals). **D** Representative neuronal tracings of CA1 pyramidal neurons (scale bar: 50 µm). **E** Quantification of total number of intersections and number of intersections/10 µm determined by Sholl analysis. **F** Total dendritic length and dendritic length/10 µm determined by Sholl analysis. **G** Number of branches and **H** branchpoints/neuron. **I** Representative of images dendrites from Ctrl and CKO mice and quantification of spine density/µm (scale bar: 100 µm). Neuronal tracings, Ctrl: n = 5, CKO: n = 5; 2 sections per animal and 5–10 neurons per section. Data are represented as mean ± S.E.M., *p < 0.05, **p < 0.01, ****p < 0.0001.

Since we previously observed an altered LTP at CA3-CA1 synapses in *Ca*_*v*_*1.2-Nex* male mice (compare: Dedic et al. [21]), we also explored LTP in female *Ca*_*v*_*1.2-Nex* animals [21]. Interestingly, CKO female mice exhibited similar LTP as compared to their Ctrl littermates, suggesting a sex-dependent effect on LTP at these synapses [student’s t-test, avg. LTP last 10 min: t_24_ = 0.03716, p = 0.9707] (Fig. 5A, B). In the same experiments, we additionally investigated paired-pulse facilitation (PPF), a type of presynaptic short-term plasticity measure, since Ca_v_1.2 was shown to be expressed in axon terminals at CA3-CA1 synapses [15]. Intriguingly, PPF in CKO mice was higher than in the Ctrl animals, indicating a lower release probability at these synapses in CKO mice, thus, providing evidence for a presynaptic function of Ca_v_1.2 [two-way ANOVA, interaction, F_(4, 120)_ = 0.6325, p = 0.6403; time, F_(4, 120)_ = 137.9, p < 0.001; genotype, F_(1, 120)_ = 26.03, p < 0.0001] (Fig. 5C).

### Disruption of Ca_v_1.2 leads to structural alterations in hippocampal pyramidal neurons

Previous in vitro studies have determined a role for Ca_v_1.2 in neuronal development. However, the role of Ca_v_1.2 on dendritic morphology is less explored. Thus, we investigated the effects of Ca_v_1.2 inactivation in glutamatergic neurons on dendritic morphology of hippocampal pyramidal neurons (Fig. 5D). Sholl analysis revealed a reduced dendritic complexity in female CKO mice, indicated by a significantly reduced number of intersections (Fig. 5E) and total dendritic length (Fig. 5F) compared to respective Ctrl littermates [two-way ANOVA-repeated measure; #intersections: interaction, F_(49, 8330)_ = 11.72, p < 0.0001; distance, F_(49, 8330)_ = 856.2, p < 0.0001; genotype, F_(1, 170)_ = 20.62, p < 0.0001; dendritic length: interaction, F_(49, 8330)_ = 10.46, p < 0.0001; distance, F_(49, 8330)_ = 837.5, p < 0.0001; genotype, F_(1, 170)_ = 26.47, p < 0.0001; student’s t-test, #intersections: t_170_ = 4.541, p < 0.0001; total dendritic length: t_170_ = 5.145, p < 0.0001]. CKO mice further exhibited a significantly reduced number of dendritic branches and branchpoints per cell compared to Ctrl animals [student’s t-test, #branches/ cell: t_171_ = 2.684, p = 0.0080; #branchpoints/ cell: t_171_ = 2.988, p = 0.0032] (Fig. 5G, H). In addition, CKO mice possessed a significantly reduced spine density compared to their Ctrl littermates [student’s t-test, t_128_ = 2.105, p = 0.0372] (Fig. 5I). Together, these data suggest a contribution of Ca_v_1.2 to different structural aspects of hippocampal pyramidal neurons.

## Discussion

The assumption that the estrous cycle would significantly influence the behavior of female rodents and introduce substantial variability has been refuted by multiple meta-analysis studies [31–33]. Nevertheless, the number of preclinical psychiatric studies exploring female genetic mouse models is still limited. This is somewhat surprising considering the well-documented sex-dependent differences in diagnosis and manifestation of psychiatric disorders, such as a higher prevalence of anxiety and mood disorders in women than in men. Accordingly, this study was designed to advance our current knowledge of the impact of Ca_v_1.2 on the behavioral and cellular levels in female mice.

Here, we demonstrate that the loss of Ca_v_1.2 in excitatory forebrain neurons differentially affects behavior and hippocampal plasticity in female *Ca*_*v*_*1.2-Nex* mice. Importantly, the detected behavioral alterations were independent of the current stage of the estrous cycle of the tested mice. In addition, we also tested the utilized *Nex-Cre* driver line and confirmed that the behavioral effects were neither influenced by the expression of Cre recombinase in forebrain glutamatergic neurons nor by the *Nex*/*Neurod6* haploinsufficiency present in heterozygous *Nex-Cre* or *Ca*_*v*_*1.2-Nex* CKO mice.

Similar to male *Ca*_*v*_*1.2-Nex* CKO mice, female CKO mice showed hyperactivity in a novel environment and increased active stress-coping behavior, while their activity in a familiar home cage was comparable to Ctrl littermates [21]. Hyperlocomotion in a novel environment and increased active coping behavior in the FST have been proposed to reflect mania-like psychomotor or arousal responses to stressors often seen in SCZ and BPD - disorders which have repeatedly been associated with CACNA1C [34–36]. Moreover, these pathologies have causally been linked to a shift in E/I balance which has also been demonstrated in other Ca_v_1.2-deficient mouse models [22, 37–40]. Interestingly, other mouse lines possessing alterations in glutamatergic circuits show similar hyperactivity in a novel environment, further supporting the hypothesis that alterations of glutamatergic circuits have the potential to convey a shift in E/I balance [34, 36, 41, 42].

Increased anxiety-related behavior and impaired cognitive performance observed in CKO mice are common symptoms across the spectrum of psychiatric disorders. Our results are consistent with previous studies that have reported increased anxiety-related behavior and cognitive impairments in male Ca_v_1.2-deficient mice [21, 23, 43, 44]. The fact that pan-neuronal Ca_v_1.2 inactivation in other studies did not result in anxiogenic behavior suggests a cell type-specific role of Ca_v_1.2 in modulating anxiety-related behaviors [21, 23, 37, 44]. The inferior performance of CKO compared to Ctrl mice in the Y-maze aligns with clinical studies that have reported working memory impairments in SCZ patients as well as in *CACNA1C* risk allele carriers [45–47]. Ctrl and CKO mice showed similar learning capabilities during the training in the MWM task and comparable long-term spatial memory (24 h after the last training trial). However, CKO mice displayed impaired remote spatial memory (7 days after the last training trial), which is consistent with previous findings in male Ca_v_1.2-deficient mice [23, 48]. These findings suggest that calcium influx through Ca_v_1.2 expressed by glutamatergic neurons is critical for reconsolidation and retrieval of remote spatial memory. Accordingly, blockade of LTCCs in dorsal hippocampal CA1 hinders reconsolidation and maintenance of long-term spatial memory, potentially through retrieval-dependent localization of protein degradation [49]. Consolidation is a protein synthesis-dependent process, and it is well established that Ca_v_1.2 is required for protein synthesis and late-phase LTP [2, 50]. In sum, these findings suggest that Ca_v_1.2 deficiency in glutamatergic neurons might impair hippocampal protein synthesis which would be required for retrieval of remote spatial memory.

In our study, different stages of the estrous cycle did not influence the observed behavioral phenotypes. While, statistically there was no interaction between genotype and estrous cycle, the behavioral phenotype pattern emerging from genotype differences was lost in the metestrous stage in anxiety and cognitive tests. One reason for this is the low number of animals per group (Ctrl and CKO) in the metestrous stage, especially in the anxiety tests. The pattern loss can also be explained by the duration of the estrous cycle and the possible capture of a transitionary phase. Estrous cycle duration in mice can last between 2 and 8 days with an average length of 4 days [51, 52]. Although there are criteria to classify the different stages according to vaginal cytology, the delineation between different stages becomes difficult when the mice are in a transitionary phase, especially for metestrous and diestrous. And based on the time of the day the samples are collected, the mice could be transitioning between the different stages. Since our study collected the samples in the afternoon, post-testing, it is possible that several animals in the two groups being in transition resulted in the loss of anxiety and cognitive behavior patterns in the metestrous stage.

The hippocampus is widely known to be essential for coding spatial working and reference memory [53]. Additionally, the role of the hippocampus in locomotion and arousal as well as emotional responses, such as anxiety, is well-documented [54–57]. Besides behavioral changes, we observed a decreased neurotransmitter release probability at CA3-CA1 synapses which is accompanied by reduced dendritic complexity and spine density in hippocampal CA1 pyramidal neurons. Previous results from our lab using male *Ca*_*v*_*1.2-Nex* mice revealed a reduced LTP in CKO animals [21]. LTP is considered to underlie mechanisms of learning and memory and involves local dendritic spine remodeling, increased spine density, and stability [58]. Pharmacological and genetic studies have linked LTP in the hippocampal CA1 region to N-methyl-D-aspartate receptor (NMDAR) activity, but there is also evidence of an NMDAR-independent, Ca_v_1.2-dependent LTP in the CA1 region which involves protein synthesis [13]. In contrast to our previously published LTP data in male CKO mice [21], we found here that Ca_v_1.2 deficiency in female mice did not affect postsynaptic LTP. Instead, evidence of a previously less-explored presynaptic function of Ca_v_1.2 emerged, i.e., CKO mice exhibited a higher paired-pulse ratio compared to Ctrl mice, suggesting that this change in short-term plasticity might contribute to the behavioral alterations observed in female CKO mice.

Besides changes in synaptic function, we identified a reduced dendritic complexity of hippocampal CA1 neurons in CKO mice compared to Ctrl littermates. To the best of our knowledge, this study is the first to unravel structural alterations of hippocampal pyramidal neurons in a Ca_v_1.2 loss-of-function mouse model. Previously, an involvement of Ca_v_1.2 in dendritic development had only been studied in gain-of-function models carrying a Timothy syndrome mutation [25]. Our current findings, together with this previous report, highlight the importance of Ca_v_1.2 in dendritic development and maturation. Expansion of pyramidal dendritic arbors during development is critical for the maturation of neuronal circuits, as post-maturation dendritic arbors are generally more stable compared to the dynamic nature of spines [58]. Furthermore, Ca_v_1.2 expression levels in mice are significantly higher during embryogenesis and gradually decrease during postnatal development and adulthood [59]. Considering the localization at dendritic shafts and spines, Ca_v_1.2 LTCCs are positioned to modulate activity-dependent signaling and activate pathways required for dendritogenesis and spinogenesis. In addition, since Cre recombination in *Ca*_*v*_*1.2-Nex* mice occurs at embryonic day E11.5, it is conceivable that excitatory neuron-specific Ca_v_1.2 deficiency impacts proper dendritic development and maturation [21]. Additionally, we observed a reduced spine density in female mice lacking Ca_v_1.2. Since spine formation is an activity-dependent dynamic process, spine loss could be a result of altered Hebbian plasticity (LTP) or homeostatic plasticity (synaptic scaling) – the two cellular mechanisms thought to be involved in spine dynamics [55, 58]. Though LTP was unaltered in our current study, CKO mice exhibited an enhanced paired-pulse facilitation which might also have an effect on spine density and dynamics. These structural and functional alterations, which are a consequence of Ca_v_1.2 deficiency in glutamatergic neurons, could represent a possible mechanism for the behavioral alterations observed in female *Ca*_*v*_*1.2-Nex* mice. However, the exact role of Ca_v_1.2 at pre- and postsynaptic locations and its involvement in spine dynamics need to be further investigated to comprehend the underlying mechanisms and their physiological consequences.

In conclusion, this study demonstrates a vital role for Ca_v_1.2 in excitatory forebrain neurons of female mice, not only at the behavioral but also at a structural and functional level. Behavioral changes are comparable to those observed in *Ca*_*v*_*1.2-Nex* males. Importantly, we uncovered sex-specific differences with regard to Ca_v_1.2-dependent synaptic function highlighting the utter importance of incorporating male and female animals in preclinical psychiatric studies. These findings set the stage for future investigations delving into signaling pathways and mechanisms involved in pathomechanisms related to psychiatric disorders.

## Supplementary information

Supplementary Figure 1
