# Supplementary material for: Cacna1c deficiency in forebrain glutamatergic neurons alters behavior and hippocampal plasticity in female mice
Source: Transl Psychiatry. 2024 Oct 6;14:421. doi: 10.1038/s41398-024-03140-2 (PMC11456591; doi:10.1038/s41398-024-03140-2)
Supplement: Supplementary file 1 — Supplementary Figure 1 [file 41398_2024_3140_MOESM1_ESM.docx]

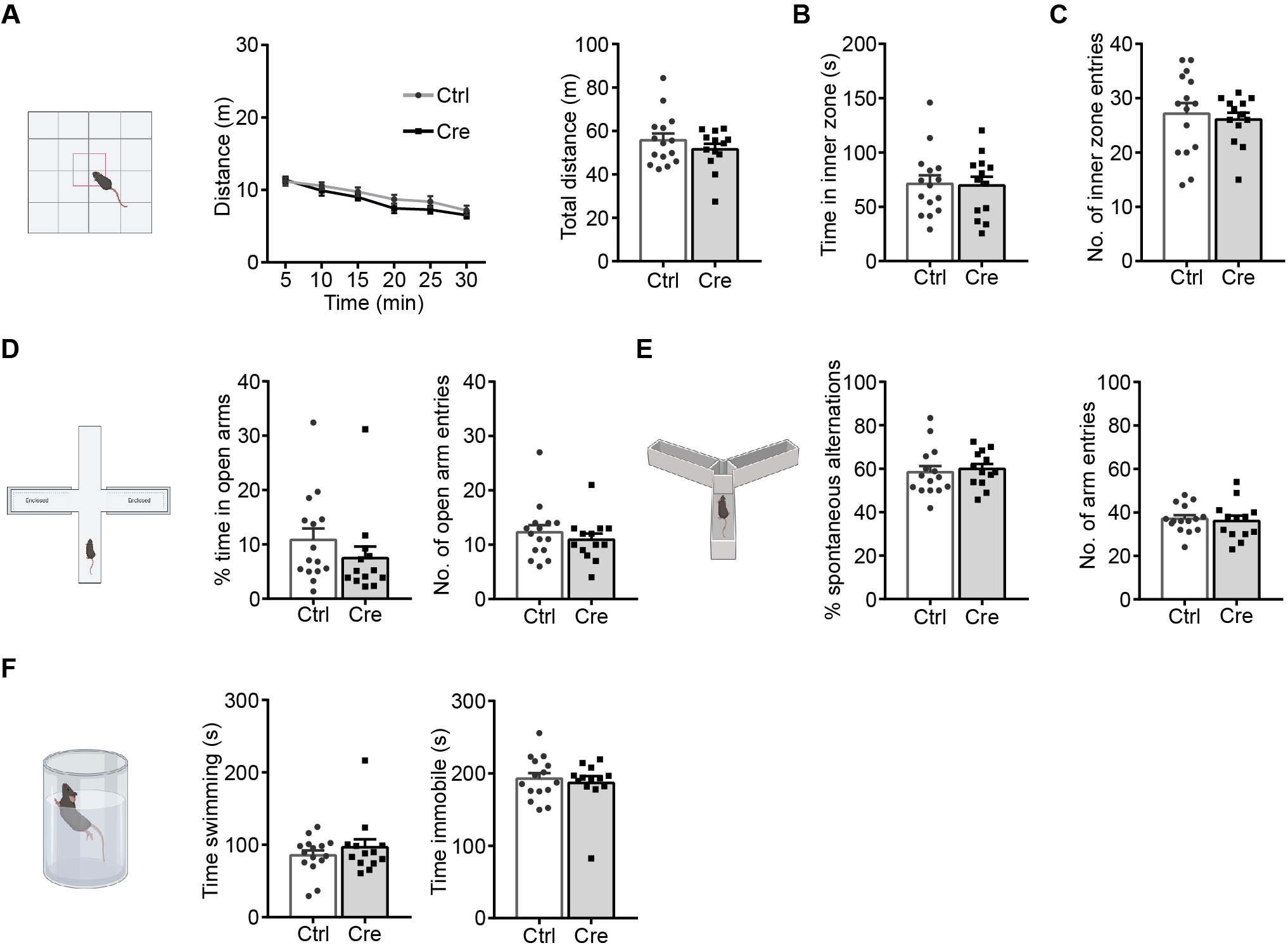


**Fig. S1 Neither *Neurod6/Nex* haploinsufficiency nor Cre expression in glutamatergic neurons affects behavior in male *Nex-Cre* mice. A** Distance travelled of *Ca_v_1.2-Nex* mice in the OFT split in 5-min time bins and total distance travelled throughout 30 min of the test [two-way ANOVA-repeated measures, locomotion: interaction, F_(5, 130)_ = 0.8719, p = 0.5020; genotype, F_(1, 26)_ = 1.044, p = 0.3164; time, F_(5, 130)_ = 36.94, p < 0.0001; student’s t-test, total distance: t_26_ = 1.022, p = 0.3164]. **B** Time spent in the inner zone during the first 5 min of the OFT and (**C**) number of inner zone entries (inner zone time: t_26_ = 0.144, p = 0.8866; inner zone entry: t_26_ =0.4365, p = 0.6661). **D** Percentage of time spent in the open arms of the EPM and number of open arm entries (student’s t-test, open arm time: t_26_ = 1.109, p = 0.2775; open arm entry: t_26_ = 0.7614, p = 0.4533). **E** Percentage of spontaneous alternations and number of arm entries in the Y-maze (student’s t-test, spontaneous alternations: t_26_ = 0.4062, p = 0.6879; arm entries: t_26_ = 0.345, p = 0.7328). **F** Time spent swimming and immobile in the FST (student’s t-test, swimming time: t_26_ = 0.8811, p = 0.3863, immobility time: t_26_ = 0.5037, p = 0.6187). (Ctrl: n = 15, Cre: n = 13). Data are represented as mean ± S.E.M..
